# Elevated Atmospheric CO_2_ Concentrations Reduce Tomato Mosaic Virus Severity in Tomato Plants

**DOI:** 10.3390/plants14050811

**Published:** 2025-03-05

**Authors:** Giovanni Marino, Andrea Carli, Antonio Raschi, Mauro Centritto, Emanuela Noris, Chiara D’Errico, Slavica Matić

**Affiliations:** 1 Institute for Sustainable Plant Protection, National Research Council of Italy (CNR-IPSP), Via Madonna del Piano 10 Sesto Fiorentino, 50019 Firenze, Italy; giovanni.marino@cnr.it (G.M.); andrea.carli@unifi.it (A.C.); mauro.centritto@cnr.it (M.C.); 2 Institute of BioEconomy, National Research Council of Italy (CNR-IBE), Via Giovanni Caproni 8, 50145 Firenze, Italy; antonio.raschi@cnr.it; 3 Institute for Sustainable Plant Protection, National Research Council of Italy (CNR-IPSP), Strada Delle Cacce 73, 10135 Torino, Italy; emanuela.noris@cnr.it

**Keywords:** CO_2_, climate change, chlorophyll content, flavonoid, nitrogen balance index, tomato, tomato mosaic virus

## Abstract

Tomato mosaic disease, caused by tomato mosaic virus (ToMV), was studied under naturally elevated [CO_2_] concentrations to simulate the potential impacts of future climate scenarios on the ToMV–tomato pathosystem. Tomato plants infected with ToMV were cultivated under two distinct [CO_2_] environments: elevated [CO_2_] (naturally enriched to approximately 1000 μmol mol^−1^) and ambient [CO_2_] (ambient atmospheric [CO_2_] of 420 μmol mol^−1^). Key parameters, including phytopathological (disease index, ToMV gene expression), growth-related (plant height, leaf area), and physiological traits (chlorophyll content, flavonoid levels, nitrogen balance index), were monitored to assess the effects of elevated [CO_2_]. Elevated [CO_2_] significantly reduced the disease index from 2.4 under ambient [CO_2_] to 1.7 under elevated [CO_2_]. Additionally, viral RNA expression was notably lower in plants grown at elevated [CO_2_] compared to those under ambient [CO_2_]. While ToMV infection led to reductions in the chlorophyll content and nitrogen balance index and an increase in the flavonoid levels under ambient [CO_2_], these physiological effects were largely mitigated under elevated [CO_2_]. Infected plants grown at elevated [CO_2_] showed values for these parameters that approached those of healthy plants grown under ambient [CO_2_]. These findings demonstrate that elevated [CO_2_] helps to mitigate the effects of tomato mosaic disease and contribute to understanding how future climate scenarios may influence the tomato–ToMV interaction and other plant–pathogen interactions.

## 1. Introduction

The atmospheric carbon dioxide concentration ([CO_2_]) has increased by approximately 50% over the past two centuries, primarily as a result of anthropogenic activities, including land use changes and the combustion of fossil fuels [1]. Recently, atmospheric [CO_2_] has reached a peak concentration of 424 μmol mol^−1^. The most pronounced rise has occurred in the past 25 years, with the levels increasing from approximately 360 μmol mol^−1^ to 424 μmol mol^−1^. This continuous buildup of atmospheric [CO_2_] is anticipated to have significant effects on plant growth and productivity [2], with wide-ranging implications for global ecosystems and agriculture [3,4,5]. Additionally, elevated [CO_2_] may influence the interactions between plant species and pathogens, potentially altering disease epidemiology and affecting crop health and yields.

Tomato (*Solanum lycopersicum* L.) serves as an important model species for the study of environmental adaptation, as it is cultivated across temperate regions and extends to high-altitude areas in tropical zones. However, the genetic diversity of cultivated tomato is notably low due to intense selective breeding and severe genetic bottlenecks during its evolution and domestication. As a result, tomatoes are particularly vulnerable to disease, with over 200 pathogens, including viruses, bacteria, fungi, and nematodes, affecting the crop worldwide during both cultivation and the post-harvest stages [6].

In the context of rising [CO_2_], pathogens can emerge as either exotic (novel) or re-emerging (native) threats. Most pathogens affecting tomato crops in the Mediterranean region, influenced by changing climatic conditions, are categorized as “re-emerging pathogens”, as they have been present in this geographical area for decades. Among these, one of the most significant pathogens is the tomato mosaic virus (ToMV), now reclassified as *Tobamovirus tomatotessellati* [7,8]. ToMV, the causative agent of tomato mosaic disease, is a highly stable and resilient virus. It remains viable in irrigation water [9] and retains its infectivity in infected seeds for up to a decade [10], posing a persistent threat to tomato production in this region.

To date, no experimental studies have simulated increases in [CO_2_] to evaluate their effects on the impact of tomato mosaic disease. To address this gap, we investigated the development of tomato mosaic virus (ToMV), a prominent pathogen in the Mediterranean region, under naturally enriched [CO_2_] conditions. The responses of plants to elevated [CO_2_] have been studied extensively using various experimental setups, including open-top chambers (OTC) and free-air CO_2_ enrichment (FACE) systems. An alternative approach is to examine plants naturally exposed to elevated [CO_2_] levels, such as those growing near natural CO_2_ springs. The Bossoleto CO_2_ spring in Rapolano Terme (Siena, Tuscany, Italy) is a unique natural laboratory that emits nearly pure CO_2_ into the surrounding atmosphere. This site has been recognized as an exceptional location for CO_2_ enrichment research [11] and has been previously utilized to study the effects of elevated [CO_2_] on photosynthesis and isoprene emissions [12]. By installing an automated irrigation system at this site and selecting a nearby control site with standard ambient [CO_2_], we were able to analyze the interaction between elevated [CO_2_] and ToMV infection in tomato plants. This experimental setup provided a unique opportunity to examine the combined effects of elevated [CO_2_] and viral infection, simulating climate change scenarios and gaining insights into the future development of tomato mosaic disease in the Mediterranean region.

## 2. Results

### 2.1. Plants Grown Under Elevated [CO_2_] Levels Show Reduced Tomato Mosaic Disease Index

**Figure 1:**
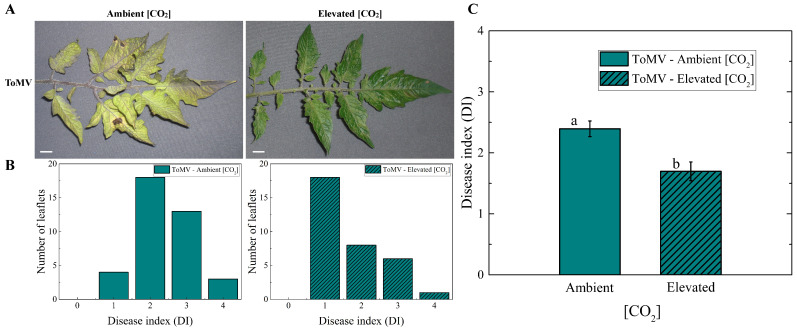
Effects of tomato mosaic virus (ToMV) infection on tomato plants at ambient and elevated [CO_2_]. (**A**) Representative images of the fourth leaf from the apexes of ToMV-inoculated tomato plants under ambient and elevated [CO_2_] conditions, photographed 40 days post-inoculation (dpi); scale bar, 1 cm. (**B**) Frequency distributions of disease index (DI) from the fourth leaf, obtained by combining values from six biological replicates, using the scoring method described in Ref. [13]. (**C**) Mean DI values under ambient and elevated [CO_2_] conditions. Error bars represent the standard error of the mean (SE). Different lowercase letters indicate statistically significant differences between groups, determined using the Kruskal–Wallis test at the 1% probability level.

Tomato plants infected with ToMV were evaluated regarding the impact of tomato mosaic disease on them under two different [CO_2_] environments: elevated [CO_2_] (naturally enriched to approximately 1000 μmol mol^−1^) and ambient [CO_2_] (ambient atmospheric [CO_2_] of 420 μmol mol^−1^). Specific symptoms of ToMV infection typically become visible on inoculated plants as early as 5 days post-inoculation (dpi). A significant reduction in disease symptoms was observed in virus-infected plants grown under elevated [CO_2_] (∼1000 μmol mol^−1^) compared to those grown under ambient [CO_2_] (∼420 μmol mol^−1^). As illustrated in Figure 1A, plants grown at ambient [CO_2_] had distinct or intense yellow and necrotic spots at 40 dpi. In contrast, the majority of the leaflets from plants grown at elevated [CO_2_] showed only slight yellowing and a few necrotic spots. To quantify the severity of virus infection, the disease index (DI) was evaluated for each leaflet of the fourth leaf from the apex using the scale described by Ref. [13], which ranges from 0 (no symptoms) to 4 (seedling death) (Figure 1B). Under ambient [CO_2_], 46% of leaflets scored a DI of 2, and 33% reached a DI of 3. In contrast, at elevated [CO_2_], the majority of leaflets (53%) scored a DI of 1. The overall disease index was significantly lower in infected plants grown under elevated [CO_2_] (1.70 ± 0.15) compared to those grown under ambient [CO_2_] (2.39 ± 0.13) (Figure 1C).

### 2.2. Elevated [CO_2_] Enhances Above-Ground Plant Growth in ToMV-Infected Tomato Plants

**Figure 2:**
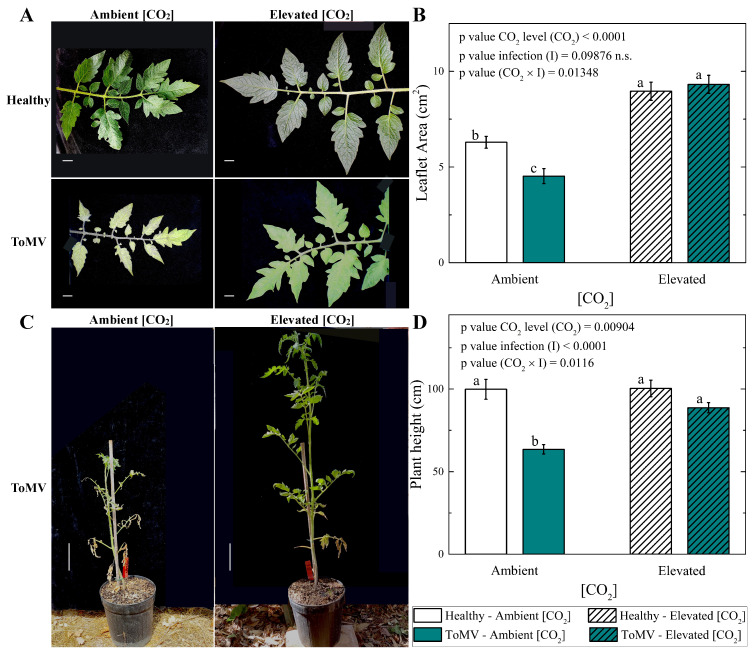
Effects of tomato mosaic virus (ToMV) infection and [CO_2_] levels on leaf size and plant height. (**A**) Representative photographs of leaves from healthy and ToMV-inoculated tomato plants grown under ambient and elevated [CO_2_] conditions, taken 40 days post-inoculation (dpi). Scale bar: 1 cm. (**B**) Mean leaflet area for healthy and ToMV-inoculated plants under ambient and elevated [CO_2_] conditions. Error bars represent the standard error of the mean (SE). Statistical significance of [CO_2_] level (CO_2_), infection (I), and their interaction (CO_2_ × I) was analyzed by two-way ANOVA. Groups with the same lowercase letters are not significantly different based on Tukey’s test at the 5% probability level. (**C**) Representative images of ToMV-inoculated tomato plants grown under ambient and elevated [CO_2_] conditions, photographed at 40 dpi. Scale bar: 10 cm. (**D**) Mean plant height for healthy and ToMV-inoculated plants under different [CO_2_] levels. Error bars represent SE. Statistical significance of [CO_2_] level (CO_2_), infection (I), and their interaction (CO_2_ × I) was determined by two-way ANOVA. Groups with the same lowercase letters are not significantly different according to Tukey’s test at the 5% probability level.

The leaf area was significantly greater in ToMV-infected plants grown under elevated [CO_2_] (∼1000 μmol mol^−1^) compared to those grown at ambient [CO_2_] (∼420 μmol mol^−1^), despite disease progression. At 40 dpi, the leaf area in infected plants grown under elevated [CO_2_] was approximately double that of plants grown under ambient [CO_2_] (9.3 ± 0.5 cm^2^ vs. 4.5 ± 0.4 cm^2^, respectively (Figure 2A,B)). A similar trend was observed in mock-inoculated (healthy) plants, where the leaf area increased by a factor of 1.4 under elevated [CO_2_] compared to ambient [CO_2_]. Notably, the leaf area in plants grown under elevated [CO_2_] reached statistically indistinguishable values regardless of their infection status, highlighting the positive impact of elevated [CO_2_] on leaf area development.

Besides the increased leaf area, faster overall growth was observed in ToMV-infected tomato plants grown under elevated [CO_2_] (∼1000 μmol mol^−1^). At 40 dpi, virus infection reduced the plant height by 0.64 times in plants grown under ambient [CO_2_] (∼420 μmol mol^−1^). Notably, under elevated [CO_2_], the plant height in infected plants increased by a factor of 1.4 compared to control plants grown at ambient [CO_2_] (Figure 2C,D). In contrast, no significant differences in plant height were observed between healthy plants grown under ambient or elevated [CO_2_], indicating that the growth-promoting effects of elevated [CO_2_] were more pronounced in ToMV-infected plants.

### 2.3. Effects of Elevated [CO_2_] on Physiological Parameters in Healthy and ToMV-Infected Tomato Plants

**Figure 3:**
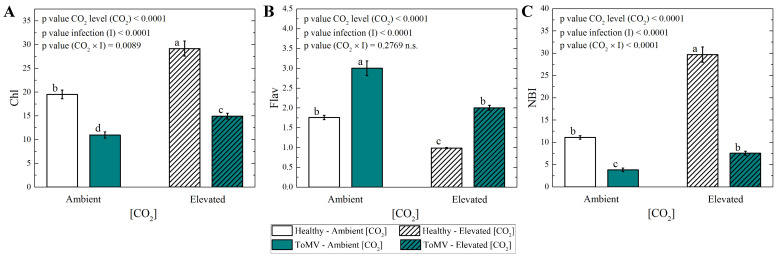
Measurements of (**A**) chlorophyll (Chl), (**B**) flavonoids (Flav) and (**C**) nitrogen balance index (NBI). Data are means of 6 plants per treatment. Error bars represent the standard error of the mean (SE). Statistical significance of [CO_2_] level (CO_2_), infection (I), and their interaction (CO_2_ × I) was analyzed by two-way ANOVA. Groups with the same lowercase letters are not significantly different according to Tukey’s test at the 5% probability level.

Elevated [CO_2_] (∼1000 μmol mol^−1^) positively influenced the chlorophyll content and the nitrogen balance index (NBI) in ToMV-infected plants. At 40 dpi, significantly higher levels of chlorophyll and NBI occurred in infected plants grown under elevated [CO_2_] compared to ambient [CO_2_] (∼420 μmol mol^−1^) (Figure 3A,C). Conversely, the flavonoid content was significantly lower in infected plants exposed to elevated [CO_2_] compared to ambient [CO_2_] (Figure 3B). A similar trend was observed in healthy plants, where the chlorophyll content, NBI, and flavonoid levels followed comparable patterns under both CO_2_ conditions.

**Figure 4:**
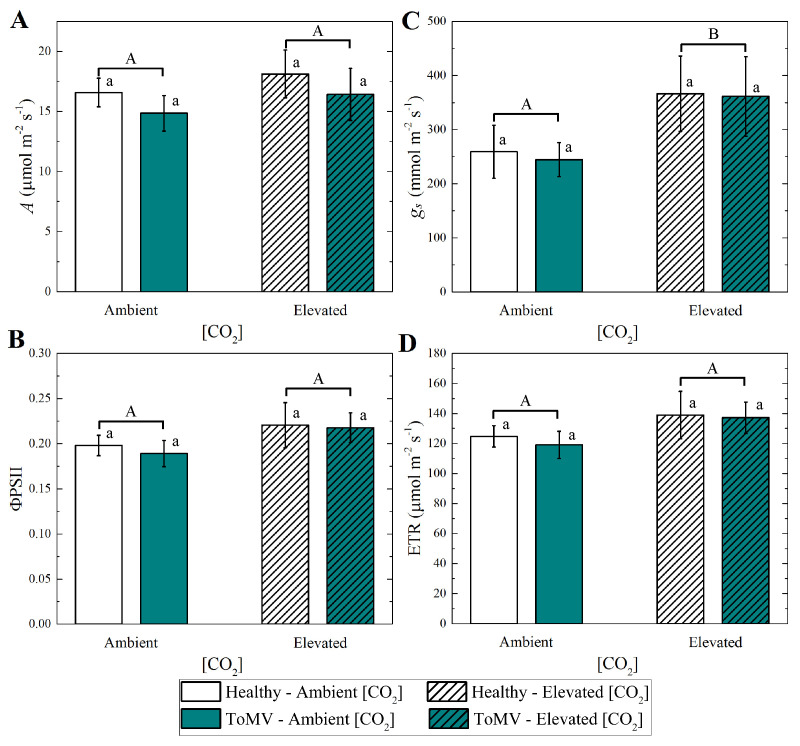
Measurements of leaf gas exchange (**A**) photosynthesis (*A*), (**B**) stomatal conductance ($g_{s}$), (**C**) fluorescence (ΦPSII), and (**D**) electron transport (ETR). Data are means of 4 plants per treatment. Error bars represent the standard error of the mean (SE). Groups with the same lowercase letters are not significantly different according to the Kruskal–Wallis test at the 5% probability level. Different uppercase letters indicate statistically significant differences in the treatments (ambient or elevated [CO_2_] levels) according to the Mann–Whitney test at the 5% probability level.

Measurements of gas exchange parameters, i.e., the photosynthesis rate (*A*), stomatal conductance ($g_{s}$), quantum yield of fluorescence (ΦPSII), and electron transport rate (ETR), showed no significant differences between healthy and ToMV-infected plants under either ambient or elevated [CO_2_] (Figure 4). This suggests that neither the adaptation to varying [CO_2_] concentrations nor the presence of ToMV symptoms significantly impacts transpiration or photosynthetic performance in newly expanded tomato leaves at 40 dpi. However, the combined effects of elevated [CO_2_] and ToMV infection led to distinct growth responses and increased chlorophyll production—in particular, enhanced stomatal conductance—for which statistical significance was observed (Figure 4B), contributing to the observed differences in the overall plant appearance and physiology.

### 2.4. Effects of Elevated [CO_2_] on ToMV Expression and Pathogenesis-Related Gene Expression in Infected Tomato Plants

**Figure 5:**
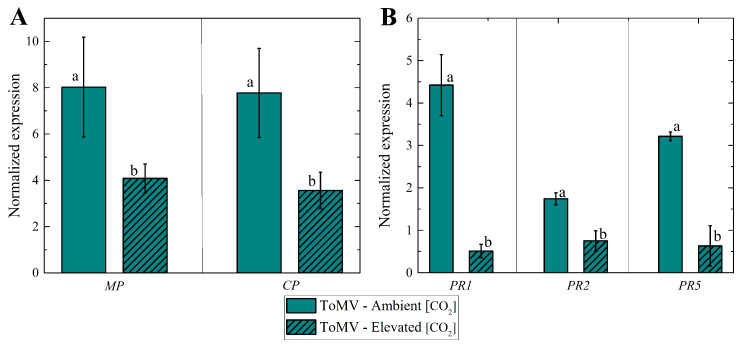
(**A**) Tomato mosaic virus expression levels of CP and MP genes at 40 days post-inoculation (dpi) under ambient and elevated [CO_2_] levels. Mean values of expression levels vs. different [CO_2_] levels; error bars represent the standard error of the mean (SE); lowercase letters indicate the statistical significance between groups, calculated using the Mann–Whitney test at a 5% probability level. (**B**) Expression levels of pathogenesis-related genes (PR1, PR2, PR5) involved in biotic stress signaling pathways in leaves from tomato plants infected with tomato mosaic virus at 40 dpi under normal and elevated [CO_2_] levels; lowercase letters indicate statistical significance between groups, calculated using Student’s t test at a 1% probability level.

In elevated [CO_2_], a slight decrease in ToMV expression for both CP and MP genes was detected in infected plants compared to those grown under ambient [CO_2_] (∼420 μmol mol^−1^), as demonstrated by two independent real-time RT-PCR assays (Figure 5A). Despite the presence of the virus, infected plants grown in elevated [CO_2_] showed excellent physiological characteristics, such as the plant height and leaf area, comparable to those of healthy plants.

Elevated [CO_2_] also significantly reduced the expression of pathogenesis-related (PR) genes, including PR1, PR2, and PR5, in ToMV-infected plants (Figure 5B). This indicates that the viral presence in plants grown under elevated [CO_2_] was not sufficiently disruptive to trigger the robust activation of PR-related genes, as observed in infected plants grown under ambient [CO_2_].

## 3. Discussion

This study provides the first detailed examination of the effects of [CO_2_] on the ToMV–tomato pathosystem, suggesting that elevated [CO_2_] mitigates the physiological stress and defense responses typically induced by ToMV infection. The Bossoleto CO_2_ spring, located in Rapolano Terme (Tuscany, Italy), served as an ideal natural laboratory for this investigation, with its ability to release nearly pure CO_2_ into the surrounding atmosphere. Equipped with an automated irrigation system, the site enabled the study of tomato mosaic disease under naturally enriched [CO_2_] conditions [11]. This unique setup facilitated the evaluation of ToMV disease development under natural, realistic environmental variables, such as the temperature, precipitation, wind, and relative humidity.

Elevated [CO_2_] plays a dual role in plant–pathogen interactions. On the one hand, it enhances photosynthesis and biomass in C3 plants like tomato, as noted in Ref. [14]. This positive effect is linked to improved nitrogen use efficiency and reduced photorespiration. However, [CO_2_] also modulates secondary metabolite production and hormonal pathways, which can alter host resistance and pathogen virulence. Ref. [15] emphasizes that elevated [CO_2_] has profound effects on plant–virus interactions by altering host physiological traits, including carbon allocation, the water use efficiency, and metabolic pathways. These changes can mitigate or exacerbate virus impacts depending on the specific pathosystem.

In addition to assessing phytopathological parameters, this study examined physiological responses, including the plant height, leaf area, and chlorophyll and flavonoid content, which are known to be affected by ToMV infection [16]. Elevated [CO_2_] significantly influenced disease progression and symptom severity, with infected plants showing notable improvements in the height and leaf area under elevated [CO_2_]. The two-way ANOVA revealed the significant effects of [CO_2_] on the flavonoid and chlorophyll content and overall photosynthetic parameters. This aligns with the findings from [14], which highlighted that elevated [CO_2_] fosters metabolic shifts that benefit plant growth and can reduce pathogen-induced stress.

### 3.1. Increased [CO_2_] Reduced the Tomato Mosaic Disease Index

Exposure to elevated [CO_2_] resulted in a reduced incidence of tomato mosaic disease caused by ToMV. These findings fit with observations from related tobamoviruses, such as tobacco mosaic virus (TMV) in tomato [17] and in tobacco and *Nicotiana glutinosa* [18]. Ref. [15] highlights that elevated [CO_2_] can reduce the severity of virus-induced symptoms by modifying the host–pathogen interaction, including altering viral replication and systemic movement. Similar reductions in disease severity under elevated [CO_2_] have also been reported in other pathosystems, including *Blumeria graminis f. sp. tritici* in wheat (1.95-fold reduction in disease index [19]), tomato yellow leaf curl virus (TYLCV) in tomato (1.15-fold reduction [20]), cucumber mosaic virus (CMV) in tobacco (1.45-fold lower viral copy number [21]), and potato virus Y (PVY) in tobacco (0.69-fold increase in yield index [22]). The 0.7-fold reduction in the ToMV disease index observed in this study is consistent with these reports.

### 3.2. Elevated [CO_2_] Promotes Above-Ground Plant Development in ToMV-Infected Tomato Plants

The increase in leaf area observed in infected plants under elevated [CO_2_] was substantial, nearly double compared to plants grown under ambient [CO_2_]. This suggests that the virus infection did not impede physiological leaf growth processes under elevated [CO_2_], as infected plants achieved leaf area values comparable to those of healthy plants. These findings are consistent with previous studies on C3 plants like tomato, which show comparable growth and photosynthetic activity at both 400 and 2000 μmol mol^−1^ of CO_2_ [23].

Furthermore, we confirmed these observations, since healthy tomato plants reached a similar height at both [CO_2_] conditions. Infected plants showed a significant increase in height (1.4-fold) under elevated [CO_2_] compared to ambient [CO_2_] and achieved heights comparable to those of healthy plants grown under elevated [CO_2_]. This indicates that the impact of ToMV on plant growth was mitigated under elevated [CO_2_]. A similar trend of a slight growth enhancement under elevated [CO_2_] was reported in zucchini plants infected with powdery mildew [24].

### 3.3. Impact of Increased [CO_2_] on Physiological Characteristics in Healthy and ToMV-Infected Tomato Plants

The chlorophyll content increased in ToMV-infected plants under elevated [CO_2_] compared to ambient [CO_2_], although it remained lower than in healthy plants. Elevated [CO_2_] is known to positively influence chlorophyll synthesis in tomato plants [20], but pathogen infection can alter crop yields. For instance, in tobacco infected by cucumber mosaic virus (CMV) or potato virus Y (PVY), the chlorophyll levels were unchanged or decreased under similar conditions [22,25]. In this study, the increased chlorophyll content in infected plants under elevated [CO_2_] suggests that the viral infection process was less detrimental to the plants’ physiological performance and chlorophyll synthesis compared to ambient [CO_2_]. This observation is in line with findings obtained in cucurbit hosts infected by powdery mildew (*Podosphaera xanthii*), where elevated [CO_2_] mitigated pathogen-induced effects [26].

The nitrogen balance index (NBI), calculated as the ratio of chlorophyll to flavonoid content, serves as an indicator of nitrogen nutrition in plants [27]. While the NBI values were consistently higher in control plants than in infected plants under both CO_2_ conditions, the NBI of infected plants grown under elevated [CO_2_] was restored to levels comparable to those of control plants grown under ambient [CO_2_]. This indicates improved nitrogen utilization efficiency in infected plants under elevated [CO_2_], as also observed under elevated [CO_2_] conditions in other pathosystems [28].

Conversely, the flavonoid content was higher in infected plants compared to healthy plants under both CO_2_ conditions, potentially reflecting the role of flavonoids as antioxidant secondary metabolites involved in signaling responses to biotic and abiotic stresses, such as ToMV infection [29,30]. However, under elevated [CO_2_], the flavonoid levels in infected plants decreased to values similar to those observed in control plants under ambient [CO_2_]. This reduction suggests that elevated [CO_2_] alleviated stress in infected plants, allowing flavonoids to be redirected toward other physiological functions [31].

### 3.4. Impact of Increased [CO_2_] on ToMV Expression and Pathogenesis-Related Gene Expression in Infected Tomato Plants

In this study, the 2-fold and 1.7-fold reductions in the relative expression of ToMV MP and CP transcripts, respectively, under elevated [CO_2_] compared to ambient [CO_2_], indicated decreased viral accumulation. This reduction in viral presence under elevated [CO_2_] correlated with a lower disease index, a renewed leaf area in infected plants comparable to that of healthy plants, and the restoration of the plant height, flavonoid content, and nitrogen balance index (NBI) in infected plants to levels similar to those of healthy plants grown under ambient [CO_2_].

The observed effective defense response against ToMV by the activation of the PR-related genes (PR1, PR2, and PR5) under ambient [CO_2_] conditions fits well with previous studies on other biotrophic pathogens, such as TMV, tomato spotted wilt virus, and *Oidium neolycopersici* [32,33,34]. The significantly lower expression of all PR genes tested in this study under elevated [CO_2_] conditions compared to ambient [CO_2_] conditions could have resulted from the lower DIs of plants infected by ToMV under elevated [CO_2_], leading to the reduced activation of these genes, which are considered markers of defense phytohormones (e.g., salicylic acid) upon infection by biotrophic pathogens [35].

## 4. Materials and Methods

### 4.1. Plant Material and Growth Conditions

Tomato plants (cv. *Marmande*) were initially grown in a greenhouse under partially controlled climatic conditions at the Institute for Sustainable Plant Protection, National Research Council (Turin, Italy), in July, with an average temperature of 23 °C during the day and 19 °C at night, average air humidity of about 60%, and natural sunlight with a day length of approximately 15 h [36]. Plants were regularly irrigated twice *per* day directly on the soil, in the cool hours of the morning and evening, to maintain soil moisture of about 70%. Each plant was cultivated in a 5 L pot filled with a substrate consisting of a sandy loam soil/expanded clay/peat mixture in a 3:2:4 ratio by volume. One month after sowing, twelve plants were mechanically inoculated at the 4-leaf stage with ToMV (p56 isolate from IPSP-CNR, Torino). To prepare the inoculum, 1 g of *Nicotiana benthamiana* leaves systemically infected with ToMV was ground in a mortar with 10 mL of inoculation buffer (20 mM Na_2_SO_3_, 10 mM Na-diethyldithiocarbamate, 5 mM EDTA). The resulting homogenate was gently applied to the upper surfaces of the third and fourth leaves of the tomato plants, using carborundum as an abrasive. Twelve mock-inoculated plants were subjected to the same procedure using a homogenate prepared from healthy leaves.

Following inoculation, in August, twelve plants (six infected and six mock-inoculated) were transferred to an elevated [CO_2_] environment (∼1000 μmol mol^−1^) at the Bossoleto CO_2_ spring in Rapolano Terme, Siena (Lat. 43.28° N, Long. 11.59° E), under natural climatic conditions [37] and systematic irrigation. The plants were exposed to these conditions for up to 40 days. Simultaneously, another group of twelve plants (six infected and six mock-inoculated) was maintained at ambient [CO_2_] (∼420 μmol mol^−1^) at a nearby control site. This experimental scheme was conducted twice during the summer seasons of 2021 and 2022, and the results reported in this study represent the average outcomes from both experiments.

### 4.2. Experimental Site

The Bossoleto natural CO_2_ spring, situated near Rapolano Terme in Tuscany, Italy, represents a unique natural laboratory for the study of elevated [CO_2_] (Supplementary Figure S1A). This site features a circular sinkhole of approximately 80 m in diameter and at least 6 m in depth [38]. The CO_2_ emissions, of geological origin and exceeding 99% purity, are released from vents located at the base and lower flanks of the cavity. These emissions create a steep vertical gradient of [CO_2_], with concentrations reaching near 100% around the vents under stable atmospheric conditions during the evening and overnight. During the day, the average long-term [CO_2_] in the upper parts of the sinkhole is approximately 1000 μmol mol^−1^, where the plants were positioned for experimentation. In addition to CO_2_, the gas emitted contains trace levels of hydrogen sulfide (H_2_S) at concentrations lower than 0.05 ppb, enhancing its value as a research site for the study of plants’ adaptation to elevated [CO_2_] [38]. A nearby site with ambient [CO_2_] (∼420 μmol mol^−1^) served as a control (Supplementary Figure S1B), where an equivalent number of tomato plants (six infected and six healthy) were maintained for comparison.

### 4.3. Infection Evaluation Through Assessment of Disease Index

Virus infection was evaluated at 40 dpi in two independent experiments conducted in 2021 and 2022 (on 9 September 2021 and 2 August 2022). The disease index (DI) was assessed on the fourth leaf from the apex in both growing conditions: ambient [CO_2_] (∼420 μmol mol^−1^) and elevated [CO_2_] (∼1000 μmol mol^−1^). The disease severity for each leaflet was ranked using the scale described in Ref. [13], with the following scores: 0 = no symptoms, 1 = slight yellowing with a few necrotic spots, 2 = distinct yellowing and necrotic spots, 3 = severe yellowing and necrotic spots with dwarfing, and 4 = seedling death.

### 4.4. Leaf Area Measurement

A Casio Exilim EX-Z85 digital camera was used to photograph the plants. Photos of the leaves used for DI evaluation were examined using a specially developed Wolfram Mathematica script (Long Hanborough, UK—Version 11.3.0.0). The leaf size was measured considering the area of the non-black region quantified based on modifications detected from RGB (red, green, and blue) values, similarly to the recent work in Ref. [34].

### 4.5. Leaf Gas Exchange and Chlorophyll Fluorescence Measurements

The photosynthetic rate (*A*) and stomatal conductance ($g_{s}$) were measured at 40 dpi in plants grown under the two experimental [CO_2_] conditions. Measurements were performed on two of the newest fully expanded leaves from each plant using a portable gas exchange system (Ciras 3, PP Systems, MA, USA). Gas exchange measurements for all treatments were conducted under ambient [CO_2_] of ∼420 μmol mol^−1^, supplied through the gas exchange system via a CO_2_ cartridge. This setup ensured comparable conditions for the evaluation of plant performance after adaptation to different [CO_2_] environments. Simultaneously, chlorophyll fluorescence parameters, including the quantum yield (ΦPSII) and electron transport rate (ETR), were measured at the same leaves using the integrated fluorometer of the Ciras 3 system.

### 4.6. Measurement of Foliar Chlorophyll, Flavonoids, and Nitrogen

A Dualex device (Dualex Research, FORCE-AR, Orsay, France) was employed to measure the adaxial and abaxial surfaces of three leaves per plant at 40 dpi. The chlorophyll index (Chl) and epidermal flavonol index (Flav) were determined following the method described in Ref. [39]. Specifically, the Chl and Flav values were calculated as the averages of the measurements obtained from both the adaxial and abaxial surfaces. The nitrogen status of the leaves was quantified using the nitrogen balance index (NBI), defined as the ratio of chlorophyll to flavonoid content, according to Ref. [27].

### 4.7. Total RNA Isolation and Real-Time RT-PCR

Total RNA was obtained from 100 mg of leaf tissue of infected and healthy plants using Trizol (Thermo Fisher Scientific, Waltham, MA, USA), followed by applying the TURBO DNase treatment (Ambion, Waltham, MA, USA) to remove DNA contamination. Total RNA (0.5 μg) was used for cDNA synthesis using a high-capacity cDNA reverse transcription kit (Applied Biosystems, Waltham, MA, USA), incubating samples at 25 °C for 10 min and at 37 °C for 2 h, followed by final enzyme deactivation at 85 °C for 5 min. The real-time RT-PCR was performed in a CFX96 Real-Time PCR instrument (Bio-Rad, Hercules, CA, USA). The reaction mixture contained 1 μL of cDNA, 1× iTaq Universal SYBR Green Supermix (Bio-Rad), and 0.25 μM of each primer. The thermal cycling conditions consisted of initial denaturation at 95 °C for 10 min and 40 cycles at 95 °C for 15 s, followed by 60 °C for 1 min. The normalization of the expression levels of tomato target transcripts was performed using the geometric mean of the elongation factor (SlEF) used as an endogenous control, following the $2^{-\Delta \Delta C_{T}}$ method [40]. Three biological replicates, each with three technical repetitions, were tested for every condition.

The same calculation was used to measure the relative expression of the ToMV coat protein (CP) and movement protein (MP) genes by real-time RT-PCR assays, as described by [9,13], respectively. The only modification was that, instead of the TaqMan real-time PCR reaction mixture, the SYBR Green real-time PCR reaction mixture was used, together with the pairs of described primers, excluding the use of the Taqman probe in both assays. The slope reaction values obtained with the SYBR Green chemistry were similar to those measured with the TaqMan chemistry, e.g., the CP reaction slope of −3.16 vs. −3. 55 [13] and the MP reaction slope of −3.23 vs. −3.56 [9]. In both SYBR Green real-time PCR assays, good primer efficiency was obtained, within the range of 90–110 % (Supplementary Figure S2). All primers used in the real-time RT-PCR experiments are listed in Table 1.

### 4.8. Statistical Analysis

The significance of the treatments (ambient or elevated [CO_2_] levels) for the DI was evaluated using the Kruskal–Wallis test at a 1% probability level (Figure 1). For datasets following a normal distribution (Figure 2 and Figure 3), the significance of the main effects, i.e., the [CO_2_] level (ambient or elevated), the infection status (healthy or ToMV-infected), and their interaction ([CO_2_] × infection), was assessed using a two-way analysis of variance (ANOVA). When the ANOVA results indicated significant effects for [CO_2_], the infection, or their interaction, Tukey’s Honestly Significant Difference (HSD) post hoc test was applied to distinguish means at the 5% probability level. For Figure 4, no statistically significant differences between groups were determined using a Kruskal–Wallis test at a 5% probability level, while statistically significant differences for the treatments (ambient or elevated [CO_2_] levels) were evaluated according to a Mann–Whitney test at a 5% probability level. For Figure 5, statistical significance was determined using a Mann–Whitney test at a 5% probability level for panel A and Student’s t-test at a 1% probability level for panel B. All statistical analyses were conducted using the OriginPro software (OriginPro 2015, Northampton, MA, USA).

## 5. Conclusions

In conclusion, this study highlights the potential of elevated atmospheric [CO_2_] to mitigate the severity of tomato mosaic disease caused by ToMV, reducing its impact on plant growth and physiological function. As the atmospheric [CO_2_] levels are projected to rise, these findings suggest a possible decrease in the detrimental effects of ToMV on tomato plants, offering an adaptive advantage under changing climatic conditions. The observed reductions in disease severity, coupled with the restoration of physiological parameters such as the plant height, leaf area, nitrogen utilization efficiency, and flavonoid content, underscore the importance of integrating these insights into agricultural management strategies. These results not only provide a deeper understanding of the ToMV–tomato pathosystem under elevated [CO_2_] but could also contribute to the development of climate-smart agricultural practices aimed at mitigating the impacts of plant pathogens. Additionally, this study lays the foundation for the examination of the broader implications of increased [CO_2_] on related tobamoviruses and other plant–pathogen systems, offering a pathway to more resilient crop production in the face of global climate change. Future studies in a controlled environment could elucidate the quantitative effects of elevated [CO_2_] on plant–virus interactions, either alone or in combination with other factors, such as rising temperatures.

## Figures and Tables

**Table 1:** List of the primers used for qRT-PCR.

Primer Name	Sequence	Ref.
SR1_*a*_4_*F*_	GTGTCCGAGAGGCCAGACTA	[41]
SR1_*a*_4_*R*_	CATTGTTGCAACGAGCCCGA	[41]
SlPR2_*F*_	TCCAGGTAGAGACAGTGGTAAA	[42]
SlPR2_*R*_	CCTAAATATGTCGCGGTTGAGA	[42]
SlPR5_*F*_	GGCCCATGTGGTCCTACAAA	[42]
SlPR5_*R*_	GGCAACATAGTTTAGCAGACCG	[42]
SlEF-fw	CTCCATTGGGTCGTTTTGCT	[43]
SlEF5-rv	GGTCACCTTGGCACCAGTTG	[43]
ToMVF	TTGCCGTGGTGGTGTGAGT	[9]
ToMVR	GACCCCAGTGTGGCTTCGT	[9]
ToMVCPf	AAAACCAGCAGAATCCGACAA	[13]
ToMVCPr	TGCAACCGTAGCGTCGTCTA	[13]

## Data Availability

Data will be made available on request.

## Supplementary Materials

The following supporting information can be downloaded at https://www.mdpi.com/article/10.3390/plants14050811/s1, Figure S1: Experimental sites used in the study. (A) Bossoleto CO_2_ spring of Rapolano Terme, a naturally rich [CO_2_] site with approximately 1000 μmol mol^−1^ [CO_2_], served as the elevated [CO_2_] experimental environment. (B) Nearby control site with ambient [CO_2_] levels (approximately 420 μmol mol^−1^), representing standard atmospheric conditions. Figure S2: Primer efficiency for ToMV Sybr-Green Real-time PCR. (A) ToMV CP primer efficiency; (B) ToMV MP primer efficiency.

## Abbreviations

The following abbreviations are used in this manuscript:

*A*	photosynthesis rate
ANOVA	analysis of variance
Chl	chlorophyll
CMV	cucumber mosaic virus
CP	coat protein
DI	disease index
dpi	days post-inoculation
ETR	electron transport rate
FACE	free-air CO_2_ enrichment
Flav	flavonoids
$g_{s}$	stomatal conductance
H_2_S	hydrogen sulfide
MP	movement protein
NBI	nitrogen balance index
OTC	open-top chamber
ΦPSII	quantum yield of fluorescence
PR	pathogenesis-related
PVY	potato virus Y
SE	standard error of the mean
ToMV	tomato mosaic virus
TMV	tobacco mosaic virus
TYLCV	tomato yellow leaf curl virus
