# Supplementary material for: Elevated Atmospheric CO2 Concentrations Reduce Tomato Mosaic Virus Severity in Tomato Plants
Source: Plants (Basel). 2025 Mar 5;14(5):811. doi: 10.3390/plants14050811 (PMC11901836; doi:10.3390/plants14050811)
Supplement: Supplementary file 1 [file plants-14-00811-s001.zip › plants-3486334-supplementary.pdf]

Supplementary Materials: Elevated atmospheric CO_2 concentrations reduce tomato mosaic virus severity in tomato plants

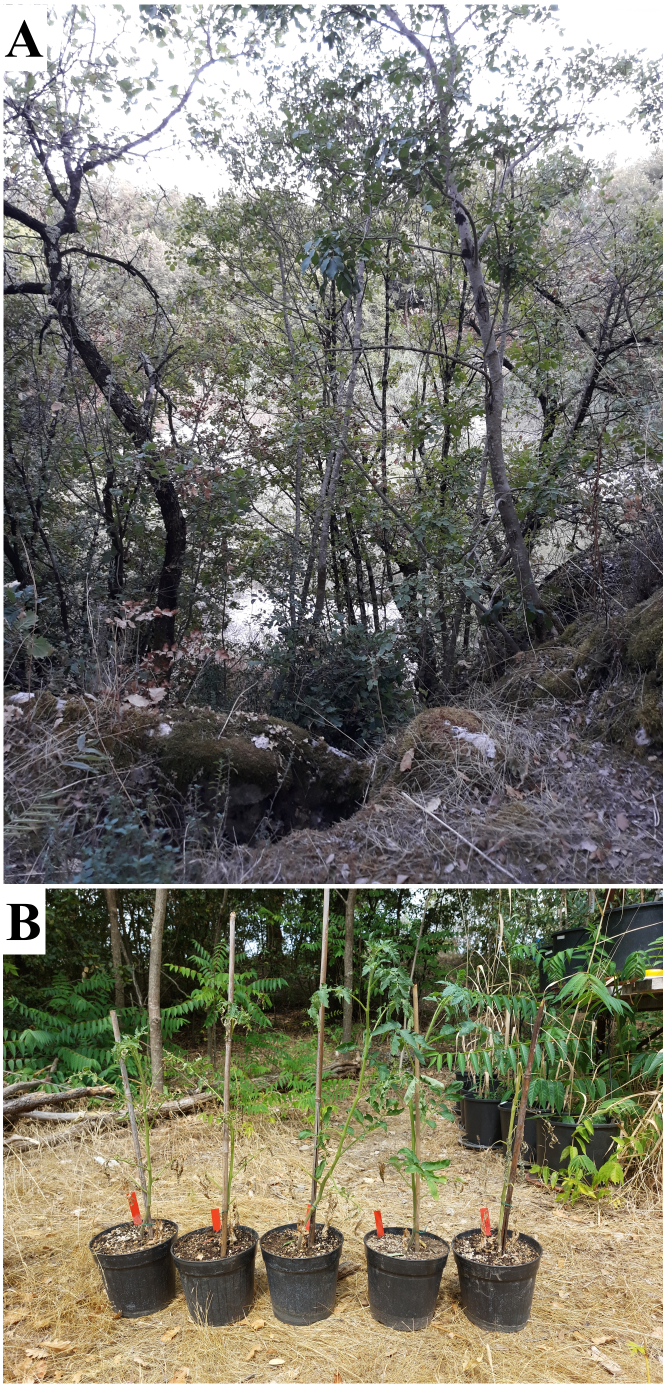

Figure S1. Experimental sites used in the study. (**A**) Bossoleto CO_2 spring of Rapolano Terme, a naturally rich [CO_2] site with approximately 1000 μmol mol^{-1} [CO_2], served as the elevated [CO_2] experimental environment. (**B**) Nearby control site with ambient [CO_2] levels (approximately 420 μmol mol^{-1}), representing standard atmospheric conditions.

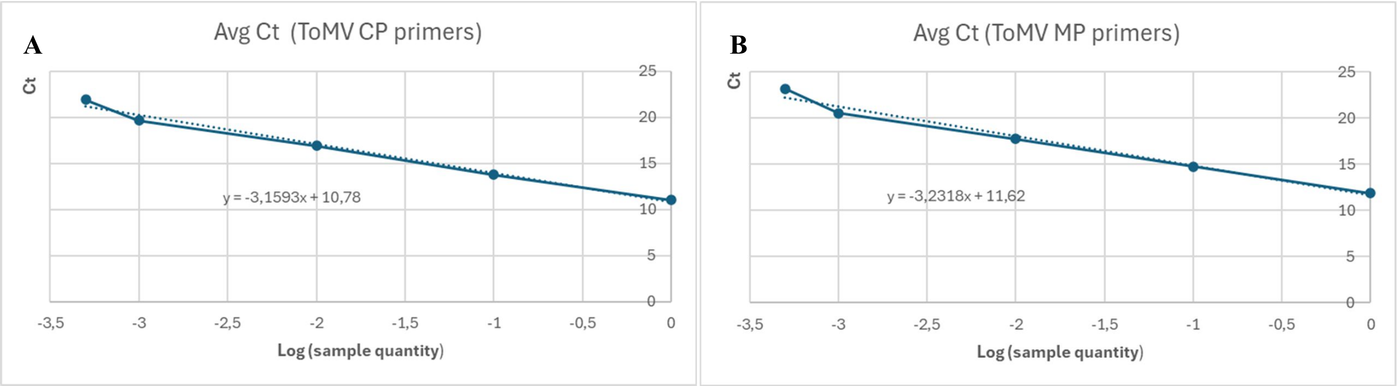

ToMV CP primer efficiency = 107,2 %

ToMV MP primer efficiency = 104,0 %

Figure S2. Primer efficiency for ToMV Sybr-Green Real-time PCR. (**A**) ToMV CP primer efficiency; (**B**) ToMV MP primer efficiency.
